# Development of 17 polymorphic microsatellite loci from Jeju striped field mouse, *Apodemus agrarius chejuensis* (Rodentia: Muridae), by 454 pyrosequencing

**DOI:** 10.1186/s41065-018-0070-8

**Published:** 2018-09-26

**Authors:** Han-Na Kim, Han-Ul Kim, Yeong-Seok Jo, Jongwoo Jung

**Affiliations:** 1 The Division of EcoCreative, Ewha Womans University, Seoul, 03760 Korea; 2 Animal Resources, National Institute of Biological Resources, Incheon, 22689 Korea; 3 Department of Science Education, Ewha Woman University, Seoul, 03760 Korea

**Keywords:** *Apodemus agrarius chejuensis*, Microsatellites, South Korea, Jeju Island, Pyrosequencing

## Abstract

**Background:**

The striped field mouse, *Apodemus agrarius*, is the most common mammal in Korea. Although microsatellite loci for the species have been identified from populations in southwestern China, amplification of those markers for Korean populations have been unsuccessful. The complicated taxonomy of Korean striped field mouse including populations on Jeju Island (*A. a chejuensis*) necessitates identification of additional molecular markers.

**Findings:**

We applied 454 pyrosequencing systems to develop a suite of microsatellite markers. Muscle tissue was harvested and sequenced from 30 Jeju striped field mouse specimens which yielded 12,165 reads with a mean length per read of 287 bp. From these reads, we identified 17 microsatellite loci for *A. a. chejuensis* and tested these new markers against samples of both *A. a chejuensis* and *A. a coreae*, the mainland taxon. All 17 loci were amplified successfully for both taxa. Of the total 17 loci, one locus failed to amplify for a population on Heuksan Island. The cross-species transferability was also tested with the allied taxon, *A. peninsulae* and confirmed successful for 12 loci.

**Conclusions:**

These newly developed markers will benefit studies of genetic structure, evolution, and resolving taxonomic problems of striped field mice and allied taxa in Korea.

## Introduction

With 24 subspecies, the striped field mouse, *Apodemus agrarius* (Pallas, 1771) is widely distributed throughout Eurasia [1]. Striped field mice are the most abundant mammal in Korea with an important place in the ecosystems as a primary prey item for predators and a vector of seed dispersal [2]. In Korea, four subspecies (*A. a mantchuricus, A. a. chejuensis, A. a. pallenscens*, and *A. a. coreae*) have been reported, but only *A. a. chejuensis* on Jeju Island has a definitive geographic barrier from the other subspecies [3]. Based on differences in morphology [4–7], physiology [8], and mitochondrial DNA [9–11], the taxonomic status of *A. a. chejuensis* has changed frequently in rank between species and subspecies. The striped field mouse is the first known vector of Hanta virus, which killed roughly 3000 United Nations soldiers per year during the Korean War, which has attracted great attention by epidemiologists [12]. Because hemorrhagic fever never occurred in Jeju Island, the island subspecies was used as a control for the isolation of Hanta virus [12].

Simple Sequence Repeats (SSRs, microsatellites) are one of the most common and efficient nuclear DNA markers for population genetics and phylogenetic studies of recently diverged taxa because they are multiallelic, highly polymorphic, abundant and show a codominant mode of inheritance [13, 14]. However, development of microsatellite markers has been costly, ineffective, and time consuming [15]. The application of next generation sequencing technology has greatly improved these challenges over the past few years [14].

To address ongoing taxonomic debates, the status of *A. a. chejuensis* and its relationship to mainland species should be examined using both haplotypic (mitochondrial DNA markers) and genotypic molecular markers (nuclear DNA markers). Wu et al. [16] developed 14 loci of microsatellite from populations in southwestern China; however, only five of 14 loci were successfully amplified in Korean striped field mouse samples [3]. Thus, nearly two thirds of the microsatellite markers available for the species failed to amplify in the targeted Korean populations [3]. Given the low transferability of the existing markers, we hypothesized that a limited proportion of markers developed for *A. a chejuensis* will be applicable for the mainland taxon, *A. a coreae* assuming their taxonomic status is different. Therefore, we developed 17 microsatellite loci from *A. a. chejuensis* for future investigation of genetic properties of the striped field mouse and then tested cross transferability for the markers against *A. a coreae* samples from the mainland.

### Sample preparation and genotyping

We used muscle tissues of *A. a. chejuensis* collected on Jeju Island to develop microsatellites loci. We collected 15 individuals each at Yongdu-dong (YD), Jeju-si (N33°31′08.72″ E126°29′47.77″) and 15 individuals at Beophwan-dong (BH), Seogwipo-si, Korea (N33°24′42.97″ E126°51′52.74″). We captured mice in Sherman traps following guidelines of the American Society of Mammalogists [17]. Specimens were deposited in the mammal collection at the National Institute of Biological Resources, Korea (NIBRMM0000105863).

Genomic DNA was extracted using DNeasy Blood & Tissue Kit (Qiagen, Valencia, CA, USA). The isolated DNA was visualized on 1% agarose gel and quantified in NanoDrop 2000 (Thermo Fisher Scientific, Waltham, MA, USA). We used a total amount of over 2 μg with concentration of 50 ng/μL or higher for 454 pyrosequencing library preparation. Library preparation, amplification, and sequencing were conducted at Macrogen Inc. (Seoul, Korea). Briefly, after DNA quality control steps, isolated genomic DNAs were fragmented using restriction enzymes and tagged them with two multiplex identifiers. After amplification of DNA fragments, the prepared libraries were pooled and sequenced in 454 GS FLX platform (Roche).

### Primer design and testing makers

Candidate loci for microsatellites were selected using QDD3 software [18]. A total of 335 primer pairs were obtained for candidate loci using PRIMER3 [19]. Each locus was then evaluated with PCR amplification performed with a total volume of 25 μl containing 1.0 μl of genomic DNA, 2.5 μl of 10× buffer (Takara), 0.7 μl of dNTP (2.5 mM each), 1.5 μl of MgCl_2_, 0.5 μl of primers each, and 0.3 U of Taq polymerase (Takara r-Taq, Takara). PCR conditions were follows: initial denaturation at 95 °C for 5 min followed by 35 cycles of denaturation at 94 °C for 60 s, annealing at 45–60 °C for 60 s, elongation at 72 °C for 90 s, and a final extension at 72 °C for 10 min. PCR products were selected and sequenced to confirm whether they contained microsatellite sequences.

A M13-tail (FAM: 5’-TTTCCCAGTCACGACGTTG-3′, VIC: 5’-GGAAACAGCTATGACCA-3′, PET: 5’-GCGGATAACAATTTCACACAGG-3′, NED: 5’-TAAAACGACGGCCAGTGC-3′) was added to the 5′ end of each forward primers while a pig tail (GTTTCTT) was added to the 5′ end of each reverse primers. For multiplex PCR, we performed PCR amplifications in a total reaction volume of 16 μl containing 5 μl of 2× QIAGEN Multiplex PCR master mix (QIAGEN), 0.08 μl of M13-tailed forward primer, 0.8 μl of pig tailed reverse primer, 0.3 μl of templated DNA and 0.16 μl of each of fluorescence primer (FAM/VIC/PET/NED). Multiplex PCR cycling conditions were as follows: initial denaturation at 95 °C for 15 min followed by denaturation at 95 °C for 30 s, annealing (14 cycles at 63 °C, 7 cycles at 58 °C, 20 cycles at 55 °C) for 90 s and 72 °C for 30 s, with a final elongation at 72 °C for 20 min.).

### Genotyping and population analysis

We genotyped 30 Jeju striped field mice (*A. a chejuensis*) from two populations (YD and BH) with the newly designed microsatellites. The amplified fragments were separated in ABI 3730xl DNA analyzer (Applied Biosystem, Inc., Forster City, CA, USA) and GeneMapper software v3.7 (Applied Biosystems) was used for manual scoring.

To test for cross-species transferability, we amplified one congener (*Agrarius peninsulae*) and one subspecies (*A. a. coreae*) using the 17 developed markers with the same PCR conditions. Additionally, we added an *A. agrarius* sample collected from Heuksan Island in the cross-species transferability run because Jo et al. [3] suggested that the *A. agrarius* population in Heuksan Island might be a newly diverged subspecies from the mainland populations (*A. a. coreae*) in Korea.

The following genetic diversity parameters were calculated using ARLEQUIN 3.5 [20]: the number of alleles at each locus (*A*), observed heterozygosity (*H*_O_), expected heterozygosity (*H*_E_) and population divergence (*F*_ST_) between the two populations (BH and YD). Polymorphic information contents (PIC) were also estimated in CENES software (http://www.ufv.br/dbg/genes/genes.htm; Cruz 2007). We tested whether significant deviation from Hardy-Weinberg equilibrium (HWE) within each population across all amplified loci in ARLEQUIN 3.5. Linkage disequilibrium (LD) between pairs of loci within each population was tested using GENEPOP 4.2.1 [21, 22]. All statistical significances of multiple comparisons were adjusted with sequential Bonferroni corrections [23]. We performed Analysis of Molecular Variance (AMOVA) to hierarchically partition the total genetic variance within and between the two populations in GENALEX v. 6.5 [24]. We also carried out principal coordinate analysis (PCoA) based on pairwise F_ST_ for all 30 individuals to examine clustering pattern among the individual genotypes in GENALEX v. 6.5 [24].

## Results & discussion

Four hundred and fifty-four pyrosequencing produced 12,165 reads with a mean length of 287 bp. We selected 335 candidate microsatellite loci from the produced reads and confirmed that 17 loci were polymorphic (Table 1).

**Table 1:** Characterization of 17 microsatellites of *Apodemus agrarius chejuensis*

Locus	Repeat motif	Repeat number range	Primer sequence (5′-3′)	Dye	Size range (bp)
AC2 L	TGTA	5–10	F: TTTCCCAGTCACGACGTTGGAACCTCAAAGAAGTGGGCTT	6FAM	213–233
			R: GTTTCTTGGGATCATCAGCAGATAGCAG		
ACH7	AACC	5–9	F: GCCCTGTACTTACCAACTCCC	6FAM	139–155
			R: GGTAGGTCAATGAGTTGGGTTC		
ACW7	CA	17–27	F: TTTCCCAGTCACGACGTTGCAAGCTTGGGATCGCAGT	6FAM	211–231
			R: GTTTCTTGGCCCTTCCTGCATACTTTG		
AC9S	CA	18–25	F: TAAAACGACGGCCAGTGCTTTGTCCTTGCAAACTACCC	6FAM	260–274
			R: GTTTCTTACCAAGATTGTAAGATGGCTGAA		
ACAQ	TG	15–31	F: TTTCCCAGTCACGACGTTGGCTTGGGATCATGTCCACTT	6FAM	162–194
			R: GTTTCTTGACTGTTTAACTTGACTCTAGATTGTG		
ACWV	CCA	6–11	F: TTTCCCAGTCACGACGTTGGCTTGGGATCAGTTGCTAGG	6FAM	316–331
			R: GTTTCTTTGTGAACACTGATGTTTATGTTCC		
ACAV	AG	5–9	F: TTTCCCAGTCACGACGTTGGGATCAAAGCCAGTCCTGAG	6FAM	192–200
			R: GTTTCTTTTCTATCTTTAGGTACTCTGTTTCCC		
ACU8	AACC	6–18	F: TTGGGATCAATCAGTCAGTCAG	HEX	291–339
			R: TGCATGAATTACTAAACAGAGTATCAA		
AC9 M	CA	4–5	F: GGAAACAGCTATGACCACCACGTTACACACACCATGC	VIC	165–167
			R: GTTTCTTGTGGTACAGGTAAAGCGTGGG		
AC124	TG	6–8	F: GGAAACAGCTATGACCATTCCCGTCTGAGTGAAGACATG	VIC	278–282
			R: GTTTCTTGCACACTCTGCTTTTTCTTGGG		
ACEC	ATGA	5–10	F: GGAAACAGCTATGACCATGAGTGCAATGGCAGAAGTC	VIC	148–168
			R: GTTTCTTGGCCTCACGAGCATACAGA		
ACQT	AG	7–12	F: GGAAACAGCTATGACCATCTTTGTTTGACACCCACCA	VIC	160–170
			R: GTTTCTTGGTTCCTCACTCCCATTCAA		
ACHZ	TTTTG	7–16	F: GCGGATAACAATTTCACACAGGTCCCTTGAAGAGTGCTCACC	PET	312–357
			R: GTTTCTTCCTGAGGAGACCCATCCTTA		
AC53	AAAC	7–15	F: GCGGATAACAATTTCACACAGGTTCAGAGGGTATACTGGGGAGG	PET	313–345
			R: GTTTCTTCAGTGTGGGCACTGTGAAAT		
ACLI	TG	5–6	F: TAAAACGACGGCCAGTGCGGCCACGCTAACAATCTCAT	NED	231–233
			R: GTTTCTTTGTTATGAATGCCCTGACCA		
AC5S	AC	20–49	F: TAAAACGACGGCCAGTGCTGGGATCAGTTAATTCAAGGC	NED	153–211
			R: GTTTCTTTCTGTAAGGCCAGAGGGCTA		
ACPJ	AC	10–36	F: TAAAACGACGGCCAGTGCTATCATCCCATAGCAGGCAGA	NED	132–184
			R: GTTTCTTAGCTTCTGTGATGGGATGGT		

F denote forward sequences and R denote revers sequences

The number of alleles per locus in the two populations of *A. a. chejuensis* varied from 2 to 11 in YD and 2–13 in BH. Ranges of *H*_E_ and *H*_O_ in YD were 0.067–0.905 and 0.067–0.867, respectively. In BH, *H*_E_ and *H*_O_ were 0.067–0.913 and 0.067–0.917, respectively (Table 2). The value of polymorphic information content ranged from 0.067 to 0.856 with a mean of 0.537 in YD. It ranged from 0.062 to 0.872 with a mean of 0.672 in BH. Three loci in YD and four loci in BH were significantly deviated from HWE after Bonferroni correction (*p <* 0.0001). No linkage disequilibrium was detected at any pair of loci.

**Table 2:** Genetic diversity indices of two populations of *Apodemus agrarius chejuensis* in Jeju Island, South Korea

			Yongdu-dong (YD)					Beophwan-dong (BH)						
Locus	n	*A*	*H*_E_	*H*_O_	*F*_IS_	PIC	HWE	n	*A*	*H*_E_	*H*_O_	*F*_IS_	PIC	HWE
AC2 L	15	5	0.605	0.200	0.677	0.543	*	15	4	0.674	0.467	0.315	0.587	ns
ACH7	15	3	0.248	0.267	−0.077	0.227	ns	15	5	0.694	0.667	0.041	0.629	ns
ACW7	15	10	0.905	0.692	0.242	0.856	ns	15	8	0.884	0.333	0.633	0.830	*
AC9S	15	7	0.779	0.800	−0.028	0.729	ns	15	7	0.867	0.667	0.237	0.814	ns
ACAQ	15	11	0.853	0.867	−0.017	0.807	ns	15	9	0.865	0.643	0.264	0.820	ns
ACWV	15	4	0.570	0.333	0.431	0.497	ns	15	1	–	–	–	–	–
ACAV	15	2	0.480	0.333	0.314	0.357	ns	15	3	0.549	0.333	0.402	0.421	ns
ACU8	15	4	0.628	0.267	0.590	0.557	ns	15	7	0.805	0.800	0.012	0.753	ns
AC9 M	15	2	0.067	0.067	0.000	0.062	ns	15	2	0.186	0.067	0.650	0.164	ns
AC124	15	3	0.191	0.067	0.659	0.175	ns	15	2	0.067	0.067	0.000	0.062	ns
ACEC	15	5	0.717	0.467	0.357	0.643	ns	15	5	0.713	0.133	0.818	0.636	*
ACQT	15	4	0.476	0.2143	0.559	0.427	ns	15	3	0.570	0.133	0.772	0.485	*
ACHZ	15	5	0.740	0.867	−0.178	0.664	ns	15	9	0.826	0.917	−0.115	0.764	ns
AC53	15	5	0.802	0.786	0.021	0.737	ns	15	8	0.870	0.917	−0.048	0.821	ns
ACLI	15	2	0.198	0.214	−0.083	0.173	ns	15	1	–	–	–	–	–
AC5S	15	8	0.885	0.467	0.473	0.824	*	15	8	0.802	0.533	0.343	0.752	ns
ACPJ	15	10	0.887	0.467	0.483	0.843	*	15	13	0.913	0.600	0.351	0.872	*

*n* number of individuals, *A* number of allele, *H*_E_ expected heterozygosity, *H*_O_ observed heterozygosity, *F*_IS_ inbreeding coefficient, *PIC* polymorphic information contents, *HWE* Hardy-Weinberg equilibrium, *ns* not significant and – uncomputed

*Significant after Bonferroni correction (*p* < 0.0029)

**Fig. 1:**
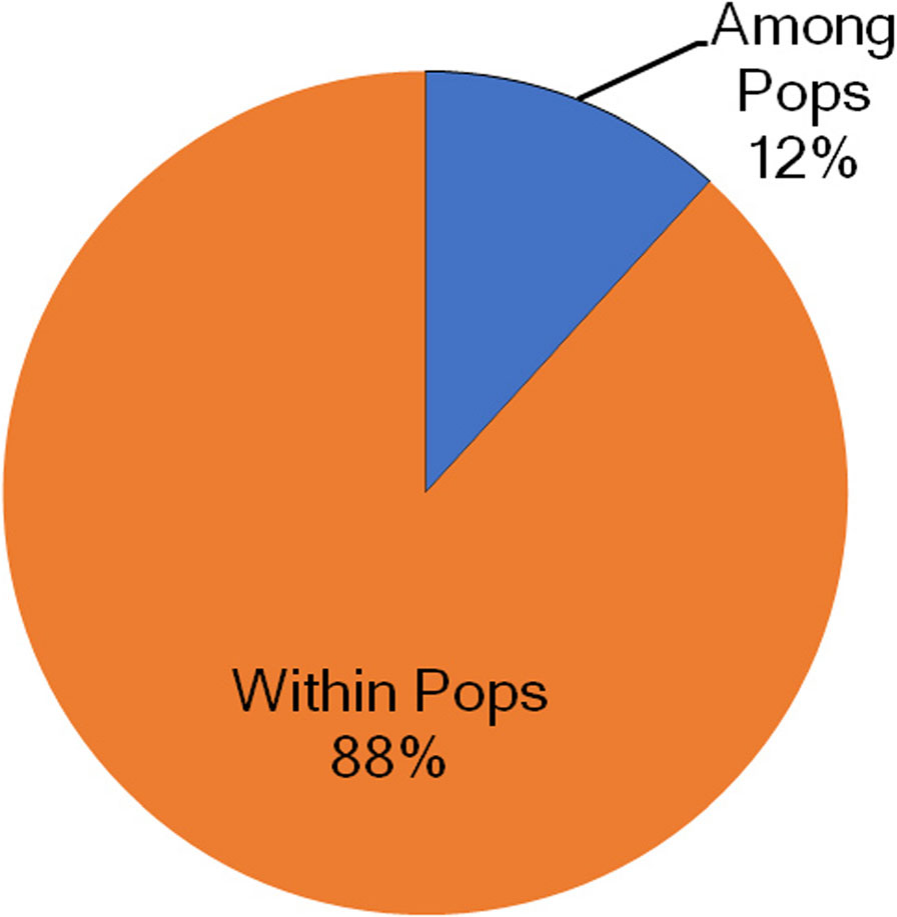
A pie chart summarizing AMOVA results. Genetic variance was hierarchically partitioned into two levels: 1) among populations (12% of total genetic variance) and 2) within each population (88% of total genetic variance)

**Fig. 2:**
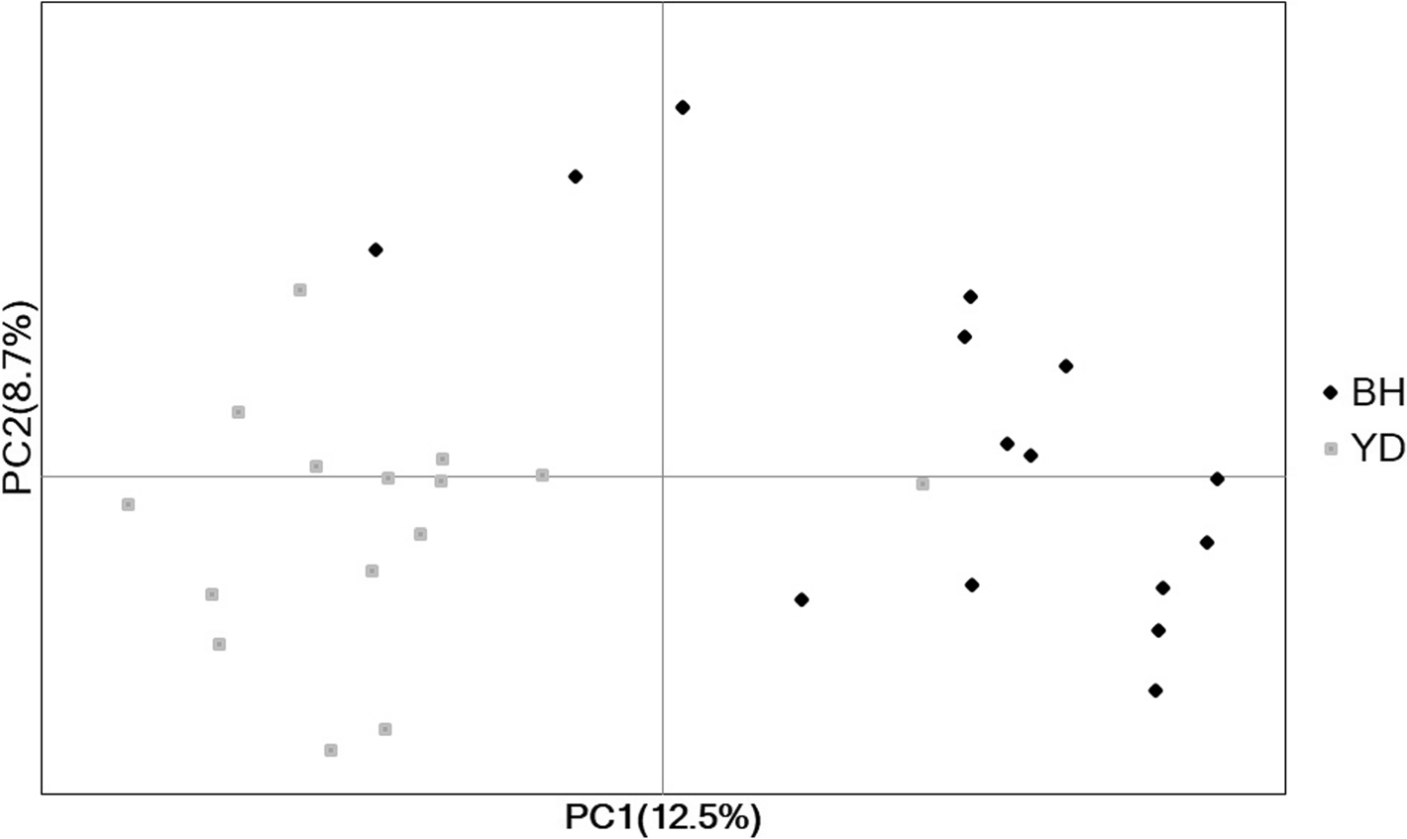
PCoA plot for 30 individual genotypes of *A. agrarius chejuensis* from two populations. The first two variance components PC1 and PC2 were plotted. BHs are samples from Beophwan-dong and YDs are from Yongdu-dong

F_ST_ (0.05) estimated between the two Jeju striped field mouse populations was not significantly different from zero (*P* > 0.99). The AMOVA results revealed that in the two populations most of genetic variation (88%) was harbored within each population, whereas among population genetic variation was proportionally minor (12%; Fig. 1). Coupled with the small F_ST_ value, the AMOVA results suggest that YD and BH are not greatly diverged in part due to high gene flow between the two populations. However, the PCoA analysis showed spatial structure among the two populations. Overall, the genotypes from each population are affiliated with the population from which the genotypes were drawn (Fig. 2). The clustering pattern indicates that the 17 markers developed from our study have sufficient power to separate out the two local populations.

Cross-species amplification examined for a subspecies, *A. a. coreae*, and its congeneric species, *A. peninsulae,* in South Korea showed high cross-species transferability. All 17 loci were successfully amplified for *A. a. coreae* and 12 of 17 loci were successfully amplified for *A. peninsulae* (Table 3). Except for the AC53 locus, all microsatellite loci were successfully amplified for individuals collected on Heuksan Island. The cross-species amplification could be an indicator of genetic distance among species. The Jeju island subspecies, *A. a chejuensis* has often been regarded a distinct species in Korea [3]. In contrast, the classification of the population on Heuksan Island has been *A. a. coreae* (the same as the mainland populations) in most of the literature despite its high genetic divergence from mainland populations as revealed in a microsatellite analysis [3]. Our cross-species amplification tests suggested that the subspecies *A. a chejuensis* might be more closely related to the mainland populations of *A. a. coreae* than the populations on Heuksan Island. Intensive population level studies with these developed markers might elucidate the true phylogenetic relationships among these three taxa.

**Table 3:** Results of cross-species amplifications

Loci	Species	
	*Apodemus agrarius coreae*	*Apodemus peninsulae*
AC2 L	+	+
ACH7	+	+
ACW7	+	+
AC9S	+	+
ACAQ	+	+
ACWV	+	–
ACAV	+	–
ACU8	+	+
AC9 M	+	+
AC124	+	+
ACEC	+	–
ACQT	+	–
ACHZ	+	+
AC53	+	+
ACLI	+	–
AC5S	+	+
ACPJ	+	+

Seven individual of each species were tested

Note: + successful PCR amplification; − PCR failed

Although this species is the main vector of Hanta virus and the most common mammal in the Korean Peninsula, few studies have addressed populations of the striped field mouse in Korea. One of the limitations in the study of *A. agrarius* is the deficiency of genetic markers [4]. The microsatellite loci developed in this study have proven to be useful for investigating genetic structure, introgression, hybrid, and taxonomic status of *A. agrarius* in not only the Korean Peninsula but the entire distribution for the species. The genetic markers might also be used to establish a management strategy for *A. a. chejuensis,* the most common endemic rodent on Jeju Island.

## Abbreviations

A: The number of allele at each locus
BH: Beophwan-dong
*H*_E_: Expected heterozygosity
*H*_O_: Observed heterozygosity
HWE: Hardy-Weinberg equilibrium
LD: Linkage disequilibrium
PIC: Polymorphic information contents
SSR: Simple Sequence Repeat
YD: Yongdu-dong
